# Early Life Stress Induced DNA Methylation of Monoamine Oxidases Leads to Depressive-Like Behavior

**DOI:** 10.3389/fcell.2020.582247

**Published:** 2020-09-08

**Authors:** Qiuyue Xu, Mingchen Jiang, Simeng Gu, Fushun Wang, Bin Yuan

**Affiliations:** ^1^School of Medicine, Nanjing University of Chinese Medicine, Nanjing, China; ^2^Department of Psychology, Jiangsu University Medical School, Zhenjiang, China; ^3^Institute of Brain and Psychological Sciences, Sichuan Normal University, Chengdu, China; ^4^Jiangsu Key Laboratory of Pediatric Respiratory Disease, Affiliated Hospital of Nanjing University of Chinese Medicine, Nanjing, China

**Keywords:** monoamine oxidase, major depressive disorder, epigenetics, DNA methylation, early life stress (ELS)

## Abstract

Major depressive disorder (MDD) is coming to be the regarded as one of the leading causes for human disabilities. Due to its complicated pathological process, the etiology is still unclear and the treatment is still targeting at the monoamine neurotransmitters. Early life stress has been known as a major cause for MDD, but how early life stress affects adult monoaminergic activity is not clear either. Recently, DNA methylation is considered to be the key mechanism of epigenetics and might play a role in early life stress induced mental illness. DNA methylation is an enzymatic covalent modification of DNA, has been one of the main epigenetic mechanisms investigated. The metabolic enzyme for the monoamine neurotransmitters, monoamine oxidases A/B (*MAO A/MAO B*) are the prime candidates for the investigation into the role of DNA methylation in mental disorders. In this review, we will review recent advances about the structure and physiological function of monoamine oxidases (MAO), brief narrative other factors include stress induced changes, early life stress, perinatal depression (PD) relationship with other epigenetic changes, such as DNA methylation, microRNA (miRNA). This review will shed light on the epigenetic changes involved in MDD, which may provide potential targets for future therapeutics in depression pathogenesis.

## Introduction

Major depressive disorder (MDD) is a major health problem and one of the leading causes of disability worldwide, and has an estimated lifetime prevalence of 16% (Gu et al., 2016). Due to its complexity, the pathology of MDD is still unclear. Recently, many studies have found that early life stress can induce long term changes in neural changes or behavioral changes. However, even though many theories suggest that the early life stress can affect the adult emotions and behaviors in a long run, but the underlying neural mechanism is far from clear. Since the etiology of MDD includes the interaction between genes and the environment, epigenetics is important for predicting utility and treatment monitoring (Webb et al., 2020). Stressful life experiences, especially early life stress, might carry out epigenetic modification of these risk genes via DNA methylation and microRNAs (miRNAs) regulation, and the expression of these genes will have long-lasting effects, which will lead to changes in brain structure and function (Pishva et al., 2017; Ding and Dai, 2019). In addition, the epigenetic differences may affect treatment response (Webb et al., 2020), however, the epigenetic mechanism of antidepressant drugs is not fully understood.

The monoamine neuromodulators have also been related to affective disorders ever since 50s in the last century and drugs targeting monoamine neuromodulators have been considered to be the first choice of treatment for these mental diseases. In addition, the monoamine neuromodulators have recently been identified as the primary neural substrates for three core affects: Dopamine-reward, Norepinephrine-stress, serotonin-punishment (Gu et al., 2019). They work together to make different basic emotions, like the three primary colors. The DA system has been proved to be involved in reward (joy), the NE has been related to the “fight or flight” (fear and anger) responses at stressful events, and the 5-HT system seems to be related to punishment (sad) (Wang et al., 2020). And dysfunctions of the monoamine system are involved in many mental disorders such as depression, post-traumatic disorders, anxiety, and attention-deficit hyperactivity disorder. Indeed, some early life events indeed induce epigenetic changes for many monoamine receptors or transporters, such as methylation DNA of MAO, or through miRNA changes. These changes would induce dysfunction of monoaminergic systems which are related to affective disorders.

Monoamine oxidase (MAO) is a major enzyme that modulates the metabolism of monoamine transmitters, including dopamine, 5-hydroxytryptamine (5-HT), norepinephrine. MAO has neurobiological origins and functions that is a potential therapeutic target in neuronal drug therapy. The *MAO A/B* genes are located on the X-chromosome (Xp11. 23) and comprise 15 exons with identical intron-exon organization, which suggests that they derived from the same ancestral gene (Shih et al., 1999). MAO regulates the levels of monoamine neurotransmitters in the brain thereby affecting signal transduction pathway and gene expression to regulate brain function, finally affecting many functions of neurons (Naoi et al., 2018). MAO catalyzed the major inactivation pathway for the monoamine neurotransmitters (Youdim and Bakhle, 2006). It is shown that *MAO A/B* act as mediators or repressors of gene expression, respectively (Inaba-Hasegawa et al., 2017; Naoi et al., 2018). *MAO A* activity fluctuates under the influence of genetic environmental factors, modulates the response of neurons to stimuli, and affects emotional and behavior activity. *MAO B* inhibitors selegiline and rasagiline can increase the expression of anti-apoptotic Bcl-2 and pro-survival neurotrophic factors in human neuroblastoma SH-SY5Y and glioblastoma U118MG cell lines Protect neurons.

Monoamine oxidase inhibitors were the first antidepressants to be developed in last century. Monoamine oxidase inhibitors increase the levels of norepinephrine, 5-HT, and dopamine by inhibiting an enzyme called monoamine oxidase (Saka, 2017). After the initial “golden age,” MAO inhibitors are currently used as third-line antidepressants (selective *MAO A* inhibitors) or clinically included as adjuvants for neurodegenerative diseases (selective *MAO B* inhibitor). However, because of its key role in regulating synapse function and monoamine metabolism, research in this field are increasing (Carradori et al., 2018). In this review, we briefly review the existing physiological data of MAO, summarize the interaction between *MAO A* and *MAO B* gene methylation and environmental factors, and discuss the pathogenesis of depression in different causes. MAO methylation mode can be used as a depression peripheral biomarker of risk and treatment responses, which might provide knowledge for future prevention and personalized treatment methods. In addition, we will provide some experiment evidence and discuss the latest advances in epigenetics and depression research. These evidences suggest that chronic unpredictable mild stress, early life stress, and perinatal stress-induced MDD vulnerability are related. Finally, we speculate future works that is needed to better understand the harmful effects of stress on MDD risk.

### Structure and Physiological Function of MAO

**FIGURE 1:**
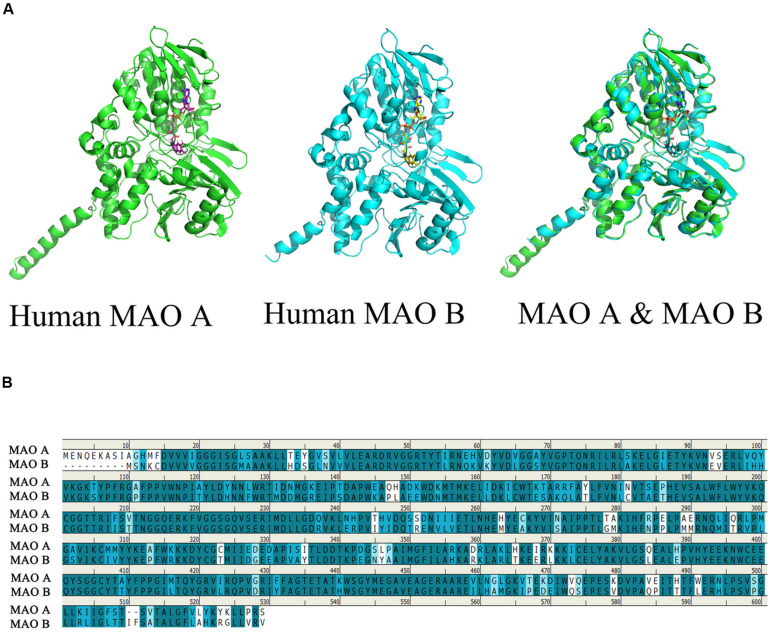
Comparison of the structure of human *MAO A* and human *MAO B*. The picture is generated by PyMOL (Son et al., 2008). Use clustalw in MEGA software to perform multiple sequence alignment, generate alignment result files, and import the aligned sequences into geneDoc software for visualization (Boratyn et al., 2019). **(A)** Observe the overlapping structure of human *MAO A* and human *MAO B* in three-dimensional space. The same part between the two structures is shown in cyan. **(B)** Amino acid sequence identity comparison, blue is the sequence with higher similarity, the darker the color, the higher the similarity, the overall sequence identity between *MAO A* and *MAO B* is about 71.1%.

Monoamine oxidases (MAOs) are mitochondrial outer membrane flavoenzymes, and are composed of 527 and 520 amino acid residues, with their molecular weights being approximately 59,700 and 58,800 for MAO A and MAOB respectively. The active forms of the isozymes *MAO A* and *MAO B* are homodimers, which are determined by their complementary DNA structures. These two isozymes are distinguished by tissue and cell distribution, substrate selectivity, inhibitor sensitivity, and separate codes (Bach et al., 1988; Ramsay, 2016). The first crystal structure of MAOs was solved in 2002 (Iacovino et al., 2018). In order to easily describe the relationship between the structure and function of this enzyme, we can use the PyMOL software, which is a molecular visualization system, to watch the structure of human *MAO A* and *MAO B*. The coordinates and the structural factors have been deposited in the Protein Data Bank, www.pdb.org (*MAO A*: PDB ID codes 2Z5X and *MAO B*: PDB code 1GOS) (Binda et al., 2004; Son et al., 2008), At the same time, we can observe the stereo view of the superposed structures of the human *MAO A* and human *MAO B* (Figure 1A).

It was found that MAO has a flavin adenine dinucleotide binding site, which is inclined to be hydrophobic aliphatic and aromatic. Therefore, the different amino acid residue binding sites in these two enzymes play a decisive role in the choice of substrates and inhibitory drugs. They catalyze the oxidative deamination of various neurotransmitters. Its main role is to catalyze the monoamines in the cell, so that the monoamines are oxidized to produce deamination. MAO acts on primary amines and their methylated secondary and tertiary amines, as well as long-chain diamines (Tipton, 2018; Sánchez-Rodríguez et al., 2020). The amino acid sequence of *MAO A* and *MAO B* can be up to 71.1% identical (Figure 1), although each enzyme has unique substrate and inhibitor specificity, *MAO A* firstly oxidizes serotonin or 5-HT and noradrenaline, whereas *MAO B* preferentially oxidizes beta-phenylethylamine (Grimsby et al., 1997). Base on the structure and physiological function of MAO, the unique position of MAO in modulating the function of a diverse series of specific neurotransmitters in association with various conditions.

### MAO DNA Methylation in Depression

DNA methylation is an epigenetic mechanism used by cells to regulate gene expression. There are many studies that reported the correlation between DNA methylation and human brain structure/function, and these studies suggest the DNA methylation can induce the following diseases: neurodevelopmental and neurodevelopmental disorders; major depression and psychosis (Wheater et al., 2020). In MDD, increased *MAO A* expression and decreased serotonin and norepinephrine brain levels are considered to be the major causative factors. Functional polymorphisms of the *MAO A* gene and genes in the serotonin signaling pathway are associated with depression. Depression in females may result from a dysregulated epigenetic programming of *MAO A*. Melas et al., have shown that females depression is related to hypomethylation in the first exon region of the *MAO A* gene. A small-scale (*n* = 44) replication study of *MAO A* methylation confirmed that female subjects with a history of depression had a hypomethylated *MAO A* compared with the control group, and the study also shows that females are hypermethylated in the same area compared with that of males (Melas and Forsell, 2015). They also found that female depressive patients showed significantly decreased methylation at ten methylated sites (CPGs) representing parts of exon I and intron I of the *MAO A* gene, compared with age-matched healthy female controls, discerned (*n* = 82–92) (Melas et al., 2013). DNA methylation levels have a major impact on depression. Interestingly, there are relatively few studies on DNA methylation of the gene encoding *MAO B*. Nevertheless, there is evidence for depression and changes in *MAO B* methylation patterns. In a sample of *N* = 199 single-egg twins without major depression, the initial association between a *MAO B* promoter CpG site and depressive symptoms could not be corrected in multiple tests (Peng et al., 2018). In fact, different environmental stress factors will affect the DNAm of different CpGs, which will affect the phenotype of depression (Table 1).

**TABLE 1:** Epigenetic changes depression: related gene and expression.

**Depression**	**DNA methylation site**	**Gene expression/affect**	**References**
CUMS	− 681 CpG	5-HT1A	Le François et al., 2015; Zurawek et al., 2019
	− 694 to −105 CpG	CRMP2	Li W. et al., 2020; Xiang et al., 2020
	− 863 to −732 CpG	Tacr2	Micale et al., 2008; Xiang et al., 2019
ELS	Hypothalamic neurons CpG	NR3C1	Bockmühl et al., 2015; Holmes et al., 2019
	chromosome 6p21.31	FKBP5	Harms et al., 2017; Tozzi et al., 2018
	CRCh37/hg19	MORC1	Nieratschker et al., 2014; Thomas et al., 2020
PD	HSD11B2	cortisol	Appleton et al., 2015; Seth et al., 2015
	−?934 CpG	OXTR	Maud et al., 2018; Tops et al., 2019
	Chromosome 3	AVP	King et al., 2017; Solomonova et al., 2019
PPD	8810078 and 8810069 CpG	Antenatal serum estradiol	Kimmel et al., 2016; Osborne et al., 2016
	− 22 to −23 CpG	NR3C1	Oberlander et al., 2008; Murgatroyd et al., 2015

### Chronic Unpredictable Mild Stress Affects Methylation

Stress is an evolutionarily adaptive response to deal with situations that impact threat to the organism and require rapid “flight or fight” responses (Wang et al., 2017). It is essential for survival and benefits to all lives, however, overwhelming stress is considered to one of the main risk factors for the development of many emotional disorders such and anxiety, depression. For example, the onset of major depression are often correlated with stressful events in previous lives, many studies reported significant correlation between the onset of major depression and the number of life altering events in the previous 3 months (Wang et al., 2017). Sometimes the previous stress happens very long ago, for example, early life stress can induce emotional depression in adult lives. This means stress can induce long term changes in the body to induce emotional disorders. Long-term or excessive stress, especially the stress generated in early life, is considered to be a high-risk environmental factor that induces various mental diseases such as depression. Elucidating the underlying molecular processes of stress-related transcriptional responses is essential for understanding the development process of stress-related mental illnesses.

It was found that patients with depressive disorder had lower *MAO A* methylation than healthy controls (Ziegler et al., 2016). Although this is not enough to explain that insufficient *MAO A* methylation can lead to depression, insufficient *MAO A* methylation may become a risk sign of mental disorders. In other words, insufficient *MAO A* methylation may be a cause of depression caused by stress. Similar to DNA modification, there are a series of different covalent modifications on RNA nucleotides encoding exotranscripts, which form gene expression by regulating RNA stability, translation and non-coding transcription functions. RNA modification, second only to epigenetic mechanisms, may represent an undescribed level of transcriptional regulation, which is highly relevant to psychiatry like MDD. Dominissini et al. found N^6^-methyladenosine (m^6^A) is the most abundant internal mRNA modification, and it is present in the entire transcriptome, at least in one-fourth of the RNA (Dominissini et al., 2012; Li Y. et al., 2020). It is found that single point mutations or expression mutations in the gene encoding neuronal glycoprotein m^6^A are associated with mental illness, and m^6^A modified genes may carry MDD risks (Du et al., 2015; Garcia et al., 2017). Researchers have used m^6^A/m sequencing (m^6^A/m-seq) and absolute quantification of transcript-specific methylation levels and found that m^6^A/m methylation in the cortex is overrepresented in genes involved in synaptic and neuronal regulation. m^6^A/m-RNA Methylation may be related to stress-induced mental illness (Engel et al., 2018).

Methylated RNA immunoprecipitation sequence analysis in the peripheral blood found that circSTAG1 is significantly reduced in chronic unpredictable mild stress (CUMS) mice and MDD patients. On the contrary, overexpression of circSTAG1 induced a positive effect on CUMS-induced astrocyte dysfunction and depression-like behavior (Huang et al., 2020). With the same detection method, the methylation level of the serotonin 1-A receptor (*5-HT1A*) promoter was found to be closely related to mRNA transcription and protein expression, the presence of stress increased the level of *5-HT1A* mRNA in the prefrontal cortex of CUMS mice by 50%. The methylation of the -681CpG site might be the main cause of *5-HT1A* transcription induced by stress (Le François et al., 2015; Albert et al., 2019).

Similarly, there are studies showing that the DNA methylation level of the collapsing response mediator protein 2 (*CRMP2*) in the hippocampus of the CUMS group was significantly higher than that of the control group, but these changes were not observed in the prefrontal cortex of CUMS rats. This indicates that the changes in *CRMP2* expression in the hippocampus and prefrontal cortex are related to the pathogenesis of depression. In addition, the results also show regional differences in the regulation of DNA methylation in the *CRMP2* promoter between the hippocampus and the prefrontal cortex during the development of depression (Xiang et al., 2020). DNA methylation is a dynamic tissue-specific event that may play an important role in the persistent and recurrent nature of depression.

### Changes Caused by Early Life Stress

**FIGURE 2:**
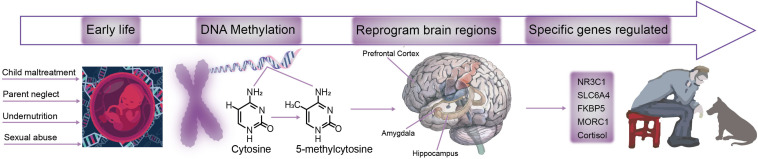
A model for the mechanism of DAN methylation affects brain function. During development, the brain is sensitive to environmental changes, such as child maltreatment, parent neglect, less nutrition, or sexual abuse. ELS induces changes in the DNA methylation-5-methylcytosine pattern, which leads to brain damages in hippocampus, prefrontal cortex, amygdala and other brain regions are reprogrammed. As a result, specific genes such as *NR3C1*, *SLC6A4*, *FKBP5*, *MORC1*, and Cortisol are impaired, and ultimately lead to an increased risk of depression and other stress-related diseases.

Early life stress is a critical causing factor for many types of mental disorders, such as depression, anxiety, posttraumatic stress (Wang et al., 2020). Many studies have found that early life stress can induce long term changes in neural changes or behavioral changes. However, even though many theories suggest that the early life stress can affect the adult emotions and behaviors in a long run, but the underlying neural mechanism is far from clear. Exposure to stress during critical periods in development can have severe long-term neural changes (Wang et al., 2020). Even though many neural and hormone changes have been suggested to underlie the changes for adult depression, but how early life stress affect adult affective disorders is still not clear. Recent epigenetic studies offer some answers for this process (Figure 2), indeed some early life events really induce epigenetic changes for many neuromodulator receptors or transporters, such as methylation DNA of MAO.

The normal development of the brain requires strict regulation of proliferation and differentiation of neural stem cells (NSCs), thereby ensuring specific number of neurons to be generated at a specific time and at a specific location (Tang et al., 2019). DNA demethylation of CpG dinucleotide sites play an important role in the cell fate characteristics of NSCs (He et al., 2020). In mouse cortex, astrocytes are mainly produced between echovirus 16 and the postpartum stage (Sauvageot and Stiles, 2002; Juliandi et al., 2010). The methylation of the Gfap promoter, a typical astrocyte marker, is anti-correlated with the expression of Gfap as same as astrocytes (Takizawa et al., 2001). DNA methylation inhibits the activation of glial production by hypomethylation of astrocyte lineage gene promoters. DNA methylation is one of the major epigenetic mechanisms that can regulate the cell fate of NSCs and control the order of neuron and glial production (Feng et al., 2007). If problems occur in the early regulatory process, the development of the brain will be affected and may lead to brain diseases.

Early life stress can “damage” the brain, leading to the possibility of developing depression later in life through epigenetic mechanisms. Early and adult stress exposure is related to epigenetic changes in genes related to mood regulation, for example, participation Genes that regulate hypothalamic-pituitary-adrenal axis activity (*NR3C1*) or genes responsible for serotonin transporter (5-HTT)-encoding gene SLC6A4 (Wang et al., 2020). Data from epigenetic studies indicate that the mechanism of action of certain antidepressants (such as fluoxetine and escitalopram) or mood stabilizers (such as valproic acid) are at least partially related to epigenetic processes (Chmielewska et al., 2019). The association between early life stress and depression is controlled by genetic risk factors, including serotonin transporter, brain derived neurotrophic factor, glucocorticoid receptor, FK506-binding protein 5 and corticotropin releasing hormone receptor 1 polymorphisms. At the same time, early life pressures will make epigenetic modifications to these risk genes through DNA methylation and miRNA regulation, which will have long-term effects on the expression of these genes, and cause changes in brain structure and function, and eventually increase Genetic susceptibility (Ding and Dai, 2019).

In recent years, the emergence of small non-coding RNAs (ncRNAs) as large controllers of gene expression has attracted attention due to their roles in various disease processes (Wen et al., 2020). Among various ncRNAs, miRNA is mostly studied, and has been regarded as the main regulator of neuroplasticity and brain function. miRNA has been proved to play a role in the maladaptive processes associated with ELS in puberty and adulthood (Wu et al., 2019). Postpartum ELS is associated with abnormal miRNA expression and function, and these processes are essential for the development of depression and suicidal behavior (Allen and Dwivedi, 2020).

### Sex Hormone and Depression

Recent studies have shown that female depression may be caused by gene and child-adversity interaction and/or *MAO A* epigenetic programming disorder (Melas et al., 2013). Observation from epidemiological data shows that the incidence of MDD in women is 2.5–3 times that of men. During puberty, the incidence of depression increases. After puberty, girls show a steeper upward trend than boys. It is also worth noting that women also experience many emotional disorders during the perinatal or menopausal period. The timing of the onset of this sex bias suggests that sex hormones contribute to depression and anxiety during puberty (Gu et al., 2018). About 20% of new mothers will develop perinatal depression (PD), which is one of the most common medical complications during and after pregnancy. The depression has short-term or long-term negative effects on mothers, children and their families. Current studies have reported many causes of PD, including genetic, epigenetic, environmental, socioeconomic and psychosocial risk factors (Hoffman, 2020). Pregnancy will induce epigenetic and other downstream changes in the maternal Oxytocin-system, which may be mediated by the action of steroid hormones. Oxytocin receptor (OXTR), a key regulator of stress and anxiety, is also affected by gonadal hormones and psychosocial risk factors. Hormonal changes cause changes in the DNA methylastion of the oxytocin gene locus promoter throughout pregnancy, affecting maternal behavior (Toepfer et al., 2019). *TTC9B* and *HP1BP3* DNA methylation during antenatal time are associated with the changes of estradiol and allopregnanolone over the course of pregnancy (Osborne et al., 2016). Estradiol levels and MAO DNA methylation also exhibited a significant interaction to associate with the ratio of allopregnanolone to progesterone (Kimmel et al., 2016).Cumulatively, the specific increased sensitivity of epigenetic reprogramming of postpartum depression (PPD) on MAO genes is confirmed, and it is indicated that epigenetic variation may be an important mediator of mood-related steroid production.

Many recent studies suggested that depression may be a reflection of chronic maternal stress and may result from glucocorticoids, all of which are related to the etiology of psychotic diseases (Stein et al., 2014). The association between stress during pregnancy and epigenetic modification of offspring DNA and the methylation of offspring DNA is also reproted (Nowak et al., 2020). Two genes are reported to be involved in stress response regulation: nuclear receptor subfamily 3 group C member 1 and 2 (*NR3C1* and *NR3C2*). DNA methylation of *NR3C1* and *NR3C2* were measured in placental and infant buccal samples, and it is found that maternal early pregnancy depressive disorder and symptoms were associated with lower DNA methylation at *NR3C2* CpG_24 in placental tissue. The changes in *NR3C2* DNA methylation levels seem to be affected by infant cortisol, which indicates that DNA methylation intervention in infancy may come from maternal hormones, and the main effect is *NR3C2* (Galbally et al., 2020). However, in childhood-adversity subtypes may differentially impact DNA methylation at *NR3C1*, baseline *MAO A* variations may affect the extent of *NR3C1* methylation (Melas et al., 2013). *MAO A* was also suggested to acts simultaneously with *NR3C2* methylation. Treatments that improve maternal depressive symptoms can reduce children’s overall DNA methylation, increase the thickness of the cortex, and reduce the cross-section of white matter fiber bundles in areas involved in cognitive function and stress response (Bleker et al., 2020).

### miRNA Relationship With DNA Methylation

Epigenetics refers to processes that affect gene expression and translation, including DNA methylation and miRNA and histone modifications. The methylation of RNA and DNA, in the form of m^6^A and 5-methylcytosine plays a vital role in various biological processes (Chen et al., 2016). DNA methylation can affect m^6^A modification by modulating the expression of m6A demethylase gene, and m^6^A demethylase feedback regulates DNA methylation, thus established a molecular relationship between 5-methylcytosine DNA methylation and m^6^A mRNA methylation during fruit ripening (Zhou et al., 2019). Further studies with human diseases found mutation frequencies of m^6^A and 5-methylcytosine regulators were increased in depression. Interestingly, the m^6^A and 5-methylcytosine regulators show a considerable level of mutation frequency in depressive patients and these two regulators also happen together (Chen et al., 2020).

DNA methylation, modification of histone and chromatin structures, and the function of ncRNAs are the core regulators for specific patterns of gene expression. Epigenetic modifiers, especially microRNAs (miRNAs), are attracting more scientific efforts because of their role in stress sensitivity after early stress. Among them, potential genetic and environmental risk factors may drive abnormal epigenetic changes to target stress response pathways, which are related to neuronal plasticity and major depression. miRNA is ∼22 nt RNA that can guide post-transcriptional inhibition of mRNA targets in a variety of eukaryotic cell lines. In humans and other mammals, these small RNAs help to sculpt the expression of most mRNAs (Bartel, 2018). Almost all brain miRNAs are co-expressed at different levels in different brain regions. There is also evidence that cell types (neurons and glial cells) specific miRNAs exist in the central nervous system, and they play a role in neuronal differentiation and synaptic plasticity.

In all, epigenetic modifiers, especially miRNAs, have received more and more scientific attention because of their role in stress sensitivity after early stress (Wigner et al., 2020). In addition to DNA methylation, other expression forms of epigenetic regulation (such as miRNA interference) may also play a key role in gene expression levels. These findings provide strong support for the argument that analysis of mRNA and protein expression levels and promoter methylation status can help understand the pathogenesis of mental illness (including depression) and the mechanism of action of drugs that effectively treat it.

## Conclusion and Discussion

Early life stress has been known as a major cause for MDD, but how early life stress affects adult monoaminergic activity is not clear either. Recently, DNA methylation is considered to be the key mechanism of epigenetics and might play a role in early life stress induced mental illness. There are many studies supporting the hypothesis that DNA methylation of *MAO A* gene might the reason for depression. Consistently, decreased methylation of *MAO A*, which may reduce monoamine utilization by increasing *MAO A* activity, has been found in depressive patients, anxiety and affective disorders. This is consistent with the monoamine depletion hypothesis of anxiety and depression, which might be the mechanism of action of *MAO A* inhibitors (such as tranylcypromine, phenelzine, or moclobemide) that are successfully used to treat depression and social phobia (Shulman et al., 2013).

In the current review, we presented evidence of epigenetic changes in depression. The effects of different pressure sources on DNA methylation were sorted out. Looking ahead, it is important to carefully study the unique mechanism by which different types of stressors alter DNA methylation. Here, we propose MAO DNA methylation as an important candidate mechanism for the onset of depression. Finally, it is very valuable to explore the differences in miRNA function due to pressure changes. Combining whole-genome expression and *in vitro* studies, these techniques can help us clarify the importance of DNA/RNA methylation in depression. It will help design a more personalized treatment plan for people with depression or suicidal behavior. As MDD involves a complexity of epigenetic regulation, and a large number of brain regions are closely related to stress-related psychopathology, studies trying to understand the interaction between ELS and epigenetics and adult neuropsychiatric diseases urgently need to improve specificity. In view of the above challenges, more advanced analytic models, including machine learning and bioinformatics, are urgently needed. Improving our understanding of epigenetic mechanisms involved in MDD can pave the way for the development of therapeutic and diagnostic interventions.

Even identical twins show substantial individual differences, which might be produced epigenetically by the two-way interaction between the brain and hormones, the immune system mediator and the autonomic nervous system (McEwen and Bulloch, 2019). Epigenetics shape the structure and function of the brain and other body systems, and the brain and body systems show considerable adaptive plasticity throughout development and adult life. As genomic research has shed lights on mechanism of depression, the etiology of mental illness is progressing from the psychodynamic origin proposed by Freud to an organic approach and epigenetic derivation.

## Author Contributions

QX, SG, and FW planned the project. QX and MJ wrote the first draft. MJ, FW, BY, and SG made major revisions to the logic of this article. All authors approved the final version of the manuscript for submission.

## Conflict of Interest

The authors declare that the research was conducted in the absence of any commercial or financial relationships that could be construed as a potential conflict of interest.

## Abbreviations

5-HT: 5-hydroxytryptamine
5-HT1A: serotonin 1-A receptor
ALKBH5: alkB homolog 5
AVP: arginine vasopressin
BDNF: brain-derived neurotrophic factor
CRMP2: collapsing response mediator protein 2
CUMS: chronic unpredictable mild stress
DNAm: DNA methylation
ELS: early life stress
FKBP5: FK506-binding protein 5
HSD11B2: Type-2 11b-hydroxysteroid dehydrogenase
m^6^A: N^6^-methyladenosine
MAO: monoamine oxidases
MDD: major depressive disorder
miRNAs: microRNAs
MORC1: microrchidia family CW-type zinc-finger 1
ncRNAs: non-coding RNAs
NR3C1: nuclear receptor subfamily 3 group C member 1
NR3C2: nuclear receptor subfamily 3 group C member 2
NSCs: neural stem cells
OXTR: oxytocin receptor
PD: perinatal depression
PPD: postpartum depression
SLC6A4: serotonin transporter-encoding gene
Tacr2: tachykinin receptor 2.
